# End-Of-Use Fly Ash as an Effective Reinforcing Filler in Green Polymer Composites

**DOI:** 10.3390/polym15163418

**Published:** 2023-08-16

**Authors:** Anastasios C. Patsidis, Manolis Souliotis

**Affiliations:** 1 Department of Materials Science, University of Patras, 26504 Patras, Greece; 2 Department of Chemical Engineering, University of Western Macedonia, 50132 Kozani, Greece

**Keywords:** green epoxy, fly ash, thermomechanical properties, dielectric behavior, energy storing/retrieving efficiency, sustainable materials

## Abstract

The aim of this study is to use fly ash powder in an environmentally friendly matrix, in a novel way, addressing environmental and disposal problems. Fly ash/epoxy composites were prepared and studied varying the filler content. An investigation of structural and morphological characteristics was conducted using of X-ray diffraction patterns and scanning electron microscopy images, which revealed the successful fabrication of composites. Thermomechanical properties were studied via dynamic mechanical analysis and static mechanical tests. The composites exhibited an improved mechanical response. Broadband dielectric spectroscopy was used to investigate the dielectric response of the composite systems over the frequency range from 10^−1^ to 10^7^ Hz and the temperature range from 30 to 160 °C. The analysis revealed the presence of three relaxation processes in the spectra of the tested systems. Interfacial polarization, the glass-to-rubber transition of the polymer matrix, and the rearrangement of polar side groups along the polymer chain are the processes that occur under a descending relaxation time. It was found that dielectric permittivity increases with filler content. Finally, the influence of filler content and the applied voltage under dc conditions was analyzed to determine the ability of the composites to store and retrieve electric energy. Fly ash improved the efficiency of the storing/retrieving energy of the composites.

## 1. Introduction

In recent years, there has been increasing interest in the development of advanced composite materials with improved properties for a wide range of engineering applications. Among these new materials, epoxy-based composites have received considerable attention due to their exceptional mechanical, thermal, and electrical properties. A particular focus in this area is on the incorporation of fly ash particles into epoxy matrices, which offer a unique opportunity to produce sustainable and high-performance composites. One of the industrial by-products produced by burning coal dust in thermal power plants is coal fly ash powder. Fly ash is made up of the fine particles removed from flue gases or exhaust using electrostatic precipitators or bag filters. They make up about 70% of the by-products of thermal power plants and are extremely difficult to handle. Millions of tons of fly ash are produced around the world every year. However, recent research has highlighted its untapped potential as a reinforcing material in composite materials [1,2,3,4,5,6]. Fly ash consists primarily of amorphous silica, alumina, and iron oxide particles, which can serve as effective reinforcements when combined with epoxy resins. The use of fly ash in composites can not only improve the properties of the resulting materials, but also provide a sustainable solution for the disposal of this waste material. The motivation for studying epoxy/fly ash composites is the pursuit of sustainable and high-performance materials. By incorporating fly ash particles into epoxy matrices, researchers can improve the mechanical properties of the resulting composites while addressing the environmental issues associated with fly ash disposal. This approach aligns with the principles of green engineering, promoting the use of waste materials for the development of value-added and eco-friendly products. By exploring recent advances in the field, researchers hope to shed light on the fabrication techniques, mechanical properties, thermal behavior, electrical conductivity and other important aspects of these composites. In addition, previous studies aimed to explore the potential applications of epoxy/fly ash composites as engineering materials in various industries such as aerospace, automotive, construction and electronics [6,7,8,9,10,11,12].

In this work, a green thermosetting epoxy resin is used as the matrix because it has high corrosion resistance, low moisture absorption, good thermomechanical behavior and high stability, and it can be processed easily, while being available at a low cost. Fly ash (FA) particles are used as the reinforcing phase. FA has several excellent properties such as the enhancement of durability [13,14], workability [15], and low shrinkage, the reduction in porosity and microcracks in the microstructure of cementitious mixtures, and the lowering of hydration reaction temperatures [16]. Moreover, the presence of FA leads to a decrease in the amount and rate of carbon dioxide (CO_2_) release [17,18,19]. To investigate the impact of fly ash particle concentration on induced dielectric properties, AC conductivity, electrical energy storage and recovery, static and dynamic mechanical behavior, and thermal properties, the prepared composites were tested via several experimental techniques varying the amounts of fly ash. Fabricated systems were structurally and morphologically characterized via X-ray diffraction (XRD) patterns and scanning electron microscopy (SEM) images. 

## 2. Materials and Methods

Fly ash/epoxy nanocomposites with filler contents of 1, 3, 5, 7, and 10 phr (parts per hundred resin per mass) were fabricated. A reference specimen of neat resin was also prepared. Entropy Resins supplied the epoxy prepolymer and curing agent marketed under the brand names “ONE Epoxy resin (High bio-based laminating epoxy)” and “ONE Resin and ONS (SLOW Hardener)”, respectively, The chemical structures of the epoxy resin and hardener are not given by the manufacturing company (Entropy Resins, North Walsham, UK) probably for commercial reasons. However, according to the datasheet, the epoxy prepolymer mostly contains the following ingredients: 4,4’-isopropylidenediphenol and oxirane, and mono[(C12-14-alkyloxy)methyl]. The main ingredients of the hardener, as indicated in the relative datasheet, are polyoxypropylenediamine, rimethylhexamethylenediamine, and methylenebiscyclohexanamine, 4,4. According to the information provided by “Entropy Resins”, more than 70% of the employed chemicals are environmentally friendly. Fly ash in the form of particles was kindly provided by Titan Cement Co., with a purity of 90–95% and size in the range of 3 to 100 μm, according to the supplier. Table 1 lists the chemical ingredients of the employed fly ash. The employed fly ash originated from the thermoelectric power plant of PPC S.A. (DEI) in Megalopolis, Greece. According to the European Standard EN197-1 [20], the Megalopolis fly ash belongs to the limestone type W, since it contains 10–15% CaO with the main ingredients being SiO_2_, Al_2_O_3_ and Fe_2_O_3_. According to the ASTM C618 standard [21], the used fly ash is of the N type, since it contains SiO_2_, Al_2_O_3_ and Fe_2_O_3_ in a percentage higher than 70%.

The following actions were taken during the preparation process. Fly ash particles were added to epoxy monomers in a pre-calculated quantity. In order to prevent the formation of clusters, the resulting mixture was slowly swirled in a sonicator at 50 °C for 10 min. Once the epoxy prepolymer and fly ash particles were thoroughly mixed, the curing agent was added at a weight ratio of 10:1 (*w*/*w*). Then, the resulting liquid underwent 15 min of magnetic agitation to further disperse the particles. The homogenized mixes were then poured into a cylindrical silicon mold and allowed to cure for a week at room temperature. Finally, the mold was placed inside an oven for 4 h for post-curing at 100 °C. In earlier research, curing and post-curing processes have been examined and established via various experimental studies [22,23,24,25].

The structural properties of the manufactured composites were determined using X-ray diffraction (XRD) patterns. Bragg–Brentano X-ray diffraction (XRD) patterns were obtained using a Bruker AXS D8 Advance (Coventry, UK) instrument. The spectral line of incident radiation was Cu Kα (l = 1.54062 Å), and the detector that was used was LynxEye. The scan mode was continuous, and the scan speed was 0.5 s per step with a 0.02° 2θ step. The source slit was 0.6 mm in width, and the current and voltage were 40 kV and 40 mA, respectively. The specimens’ morphology was examined via scanning electron microscopy (SEM) by employing a Carl Zeiss EVO MA 10 device. With the same setup, the composition of the systems and the mapping of ingredients was conducted via energy-dispersive X-ray spectroscopy. To conduct the dynamic mechanical analysis (DMA) tests, TA Instruments’ TA Q800 equipment was used to evaluate dynamic mechanical behavior. The performed experiment was a three-point bending test, with applied temperatures between ambient temperature and 100 °C, at 1 Hz for the oscillating mechanical stimulus. The prepared composites’ static mechanical properties were examined using an Instron 5582 tester at room temperature at a tension rate of 5 mm/min. Broadband dielectric spectroscopy (BDS) was used to analyze the dielectric properties of the manufactured composites using Alpha-N Frequency Response Analyzer, which was purchased from Novocontrol Technologies GmbH & Co. KG (Montabaur, Germany). 

The frequency of the applied AC field ranged between 0.1 Hz and 10 MHz at a constant Vrms of 1 V. The temperature was controlled using a Novotherm system (Novocontrol Technologies, Montabaur, Germany) with 0.1 °C accuracy. The employed dielectric cell (BDS 1200, Novocontrol Technologies) was a set up with two gold-plated metal electrodes between which samples were placed in a parallel-plate capacitor configuration. All samples were tested under isothermal conditions, scanning the frequency of the applied field, from 30 to 160 °C. The used temperature step between successive scans was 5 °C. The data recordings were automatically and continuously acquired through the use of WinDETA software (Windows 10, 64 bit version).

AC dielectric testing was performed in accordance with ASTM D150 standards [26]. A high-resistance meter (DC, Agilent 4339B, Agilent Technologies, Santa Clara, CA, USA) was used to perform the DC electrical measurements. In order to maintain continuous control over the charging/discharging sequence, in the experimental setup an automatic measurement process was integrated. An appropriate software, created in the Smart Materials & Nanodielectrics Laboratory, was used for automatic and real-time data recording [24,25]. Specimens were sandwiched between a pair of parallel electrode plates in the testing cell. Each specimen was tested at two charging levels, namely 100 and 200 V, for a period of 60 s. The charging procedure was followed by a 300 s discharging one. Naturally, during the discharging procedure no voltage was applied to the specimens. A discharging short-circuit procedure was used in advance of each measuring sequence to ensure that the specimens were free of any residual charges. All DC tests were performed in accordance with ASTM D257 standards [27] in the Smart Materials & Nanodielectrics Laboratory of University of Patras, Greece. In [28], the experimental design and methodology used are described in detail.

## 3. Results

**Figure 1:**
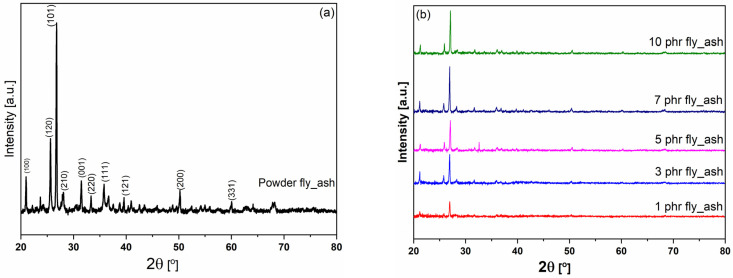
XRD patterns of the as-received fly ash particles (**a**) and the fabricated epoxy/fly ash composites (**b**).

The XRD diffractographs of fly ash/epoxy composites at various filler concentrations are shown in Figure 1, along with the XRD pattern of fly ash powder. No diffraction peaks can be seen in the XRD graph of the amorphous polymer matrix. Therefore, all the peaks seen in the nanocomposites’ patterns originate from the fly ash filler, proving that the particles were successfully dispersed throughout the epoxy. The recorded peaks are in accordance with those reported previous studies on the same type of fly ash [29]. In addition, as predicted, the filler’s concentration increases the intensity of the peaks.

**Figure 2:**
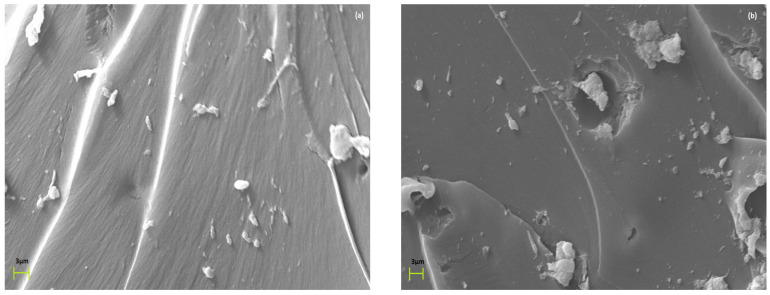
SEM images for the (**a**) 1 phr fly ash/epoxy and (**b**) 5 phr fly ash/epoxy composites.

SEM images of Figure 2 provide evidence of the morphology of the fabricated composites. From the obtained images, it can be concluded that fly ash microparticles are finely dispersed in the matrix and no extensive clusters are detected. With an increase in filler content, a limited number of agglomerates can be observed. Figures S1–S3 depict the composition of the composites and the mapping of the fly ash ingredients for the 1 phr/epoxy and 5 phr/epoxy specimens, as obtained via energy-dispersive X-ray spectroscopy. Figure S4 presents an overall image of the composite with the higher fly ash content (10 phr) at lower magnification, verifying the fine dispersion of the inclusions. 

**Figure 3:**
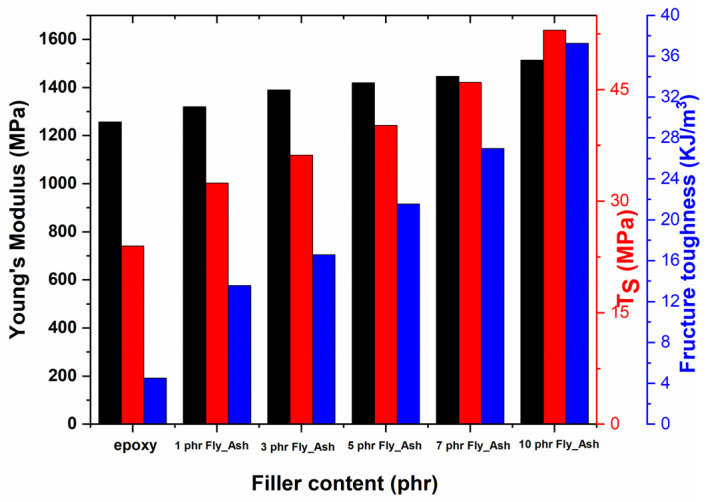
Young’s modulus, tensile strength, and fracture toughness as a function of fly ash content.

Figure 3 displays the outcomes of the static mechanical tests. Tensile stress–strain plots were used to derive values of Young’s modulus, tensile strength, and fracture toughness for each system. Elastic modulus steadily rises with the percentage of fly ash added to a material. Filler content has a significant impact on tensile strength and fracture toughness, elucidating the reinforcing ability of fly ash on mechanical properties and the strong adhesion of reinforcing particles with the matrix. At higher filler contents, the values of tensile strength and fracture toughness increase by multiple times with respect to the unfilled polymer matrix. In general, fly ash increases the mechanical durability of nanocomposites, and the employed particles do not act as stress concentration points or stress raisers within the epoxy matrix. The latter should lead to a considerable decrease in both tensile strength and fracture toughness with filler content. This seems to be consistent with the results of DMA testing.

**Figure 4:**
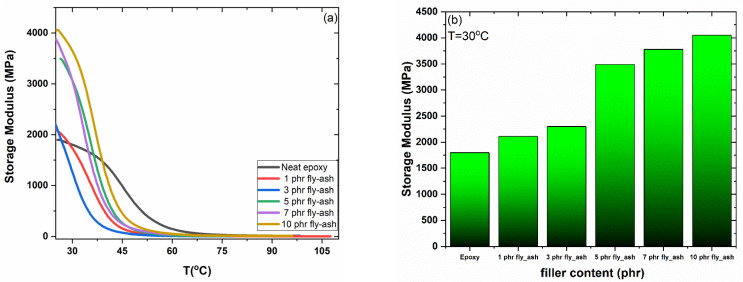
(**a**) Storage modulus as a function of temperature and (**b**) maximum values of storage modulus as a function of filler content, for all studied systems.

Figure 4 depicts the thermomechanical behavior of all the systems studied as determined via DMA data. The effect of temperature and the percentage of the reinforcing phase on the storage modulus is depicted in Figure 4a. As the temperature rises, the polymeric matrix changes from a rigid glassy state to a viscous rubber- one, as evidenced by the decrease in all the curves. Storage modulus values increase systematically with fly ash content, demonstrating the reinforcing ability of metal oxide particles in the glassy state.

The maximum values of the storage modulus as a function of fly ash content are plotted as a bar graph in Figure 4b, further elucidating this behavior. As the amount of reinforcing phase particles increases, the maximum values of the storage modulus rise, which can be considered a strong indication of the particles’ fine dispersion [30]. The results of the DMA tests agree with those of the static mechanical analysis presented earlier.

**Figure 5:**
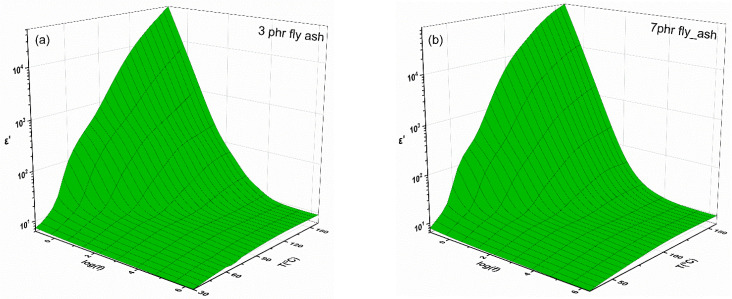
Real part of dielectric permittivity (ε′) as a function of frequency and temperature for the composites with (**a**) 3 phr and (**b**) 7 phr of fly ash content.

**Figure 6:**
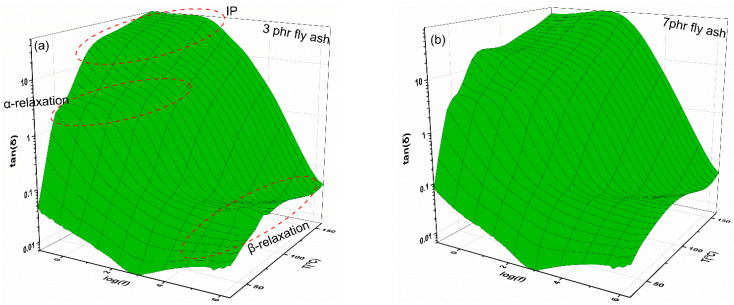
Loss tangent (tanδ) as a function of frequency and temperature for the composites with (**a**) 3 phr and (**b**) 7 phr of fly ash content.

Due to their free-charge carrier’s low density, polymer composites are classified as electrical insulators; as such, their electrical properties should be related to dielectric relaxation phenomena that occur under AC conditions. The relaxation processes, resulting from the orientation of the permanent and induced dipoles, are associated with the space charge’s migration and the presence of dipolar groups in the polymeric chains. Using three-dimensional graphs of the real part of dielectric permittivity (ε′) and loss tangent (tan δ) as functions of frequency and temperature, Figure 5 and Figure 6 show the dielectric response of the nanocomposite systems with 3 phr of and 7 phr of fly ash, as the reinforcing phase. 

The temperature and frequency dependence of the real part of the dielectric permittivity is shown in Figure 5a,b. The real part of dielectric permittivity drops with increasing frequency. Low ε′ values are caused by the inertia of the dipoles, both permanent and induced, to follow the alternating applied field at high frequencies [31]. Since dipoles have more time to orient themselves in the direction of the field, ε′ values rise in the low-frequency region. The thermal activation of the dipoles also supports the polarization process, so the maximum values of ε′ are observed at low frequencies and high temperatures. The 3D spectra show evidence of relaxation processes in the form of two step-like transitions.

Figure 6 shows 3D loss tangent spectra, which provide a clearer picture of these processes. The loss tangent (δ) is plotted against temperature and frequency in Figure 6 for the same samples as those in Figure 5. The recorded three peaks in the spectrum of dielectric loss can be interpreted as evidence of different relaxation processes. In particular, large dipoles are formed at the interface between the epoxy resin and the fly ash inclusions, because of the accumulation of unbounded charges. The effect is amplified when the temperature and the frequency are high and low, respectively. The large size of these dipoles gives them increased inertia, so their alignment with the applied field requires a longer time and thermal agitation, resulting in high permittivity values in this region. Interfacial polarization (IP) describes this type of polarization process. The process taking place in the intermediate zone is a reflection of the glass-to-rubber relaxation of the epoxy matrix (α-relaxation).

During this process, macromolecules relax in a cooperative manner, because of the cross-links, and thus large portions of the polymer chains are able to be aligned with the external field via performing cooperative segmental motions. The third relaxation, β-relaxation, is observed in the high-frequency region and is ascribed to the realignment of small polar side groups of the polymer chain. β-relaxation is a weak secondary process. The dielectric behavior shown in Figure 5 and Figure 6 is indicative of all studied systems. Figures S4 and S5 present the dielectric response of the unfilled epoxy resin and of the composite with 5 phr of fly ash. A common general performance it is apparent. 

**Figure 7:**
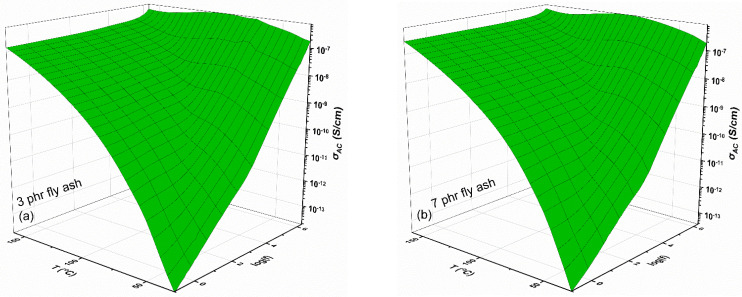
AC conductivity as a function of frequency and temperature for the composites with (**a**) 3 phr and (**b**) 7 phr fly of ash content.

Figure 7 is a three-dimensional representation of the AC conductivity of the same two composites as a function of frequency and temperature. The spectra demonstrate that the AC conductivity varies with both frequency and temperature. The higher impact of temperature in the low-frequency range indicates that the conduction mechanism is affected by temperature. The investigated systems consist of an insulating matrix and semiconducting inclusions; therefore, it is plausible that they exhibit low conductivity values that increase with temperature. At low frequencies, the applied field alternates slowly, allowing charge carriers to cover greater distances. However, due to the existence of the insulating matrix, which creates high potential barriers, this mobility is restricted, even though a small number of carriers participate in the process and thermal activation helps them to overcome some of these barriers, leading to an increase in conductivity with temperature [32]. The exponential frequency dependence of AC conductivity at high frequencies is temperature-independent. When the field is highly alternating, carriers can only “hop”/move from one adjacent site to another adjacent one, separated by low potential barriers. This eliminates the need for thermal activation and results in a dramatic increase in the number of charge carriers involved in the process, despite a significant decrease in the distances that those carriers could travel. The occurring conduction process is known as hopping conduction and refers to the movement of charge carriers such as electrons, ions, and polarons [32]. The universal law of AC conductivity [33], valid for disordered systems [34], describes the frequency-dependent change in AC conductivity at a constant temperature:

(1) $$\sigma_{AC}\left(\omega \right)=\sigma_{DC}+A{\left(\omega \right)}^{s}$$

where *σ_DC_* is the DC limiting value of conductivity, *ω* is the field’s angular frequency and *A* and *s* are temperature- and filler-dependent parameters. Figure 7 demonstrates how temperature causes an upward shift, in the frequency range, of the exponential portion of the AC curves. The composites’ relaxation mechanisms account for the formation of ‘shoulders-like’ peaks in the intermediate frequency range.

**Figure 8:**
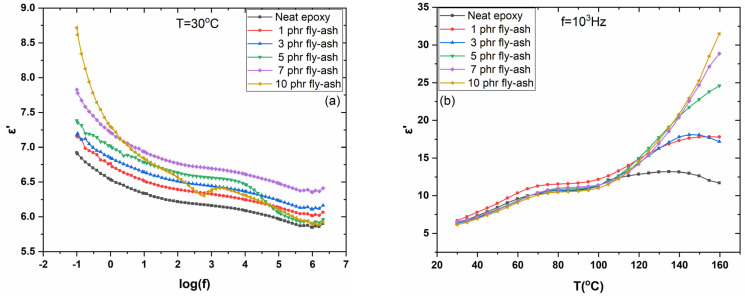
(**a**) Real part of dielectric permittivity (ε′), at 30 °C, as a function of frequency, and (**b**) real part of dielectric permittivity (ε′), at 10^3^ Hz, as a function of temperature.

Figure 8a shows the real part of dielectric permittivity against frequency for each of the examined systems at 30 °C. The increase in ε′ with the filler content reveals an additional aspect of the reinforcing ability of the fly ash particles. When compared to neat epoxy, all reinforced systems show greater values of ε′. In addition, it is well-observed that ε′ values decrease as the frequency increases because of the reduction in polarization. In Figure 8b, the variation of the real part of dielectric permittivity versus temperature, at constant frequency of 1 kHz, for all studied systems can be seen. The gradual increase in ε′ in the temperature range between approximately 40 and 80 °C is related to the facilitation of dipole orientation in the region where glass-to-rubber transition takes place. The more intense increase in ε′ values above 120 °C is attributed to IP, which occurs at high temperatures and is dependent on the heterogeneity of the composites [31,35]. Apparently, the real part of dielectric permittivity increases with fly ash content as a result of the enhancement of interfacial polarization because of the increase in the systems’ heterogeneity. Dielectric permittivity is greater in the fly ash-reinforced samples than in the insulating matrix. For this reason, the reinforced systems achieve greater values of ε′ across the entire temperature and frequency spectra.

Dielectric data can be analyzed via different formalisms, that is, dielectric permittivity, AC conductivity and electric modulus. Electric modulus eliminates the parasitic effect of electrode polarization and all relevant capacitances and is defined as the inverse quantity of complex permittivity, as shown by Equation (2):

(2) $$M^{*}=\frac{1}{\varepsilon^{*}}=\frac{1}{\varepsilon^{\prime }-j\varepsilon^{\prime \prime }}=\frac{\varepsilon^{\prime }}{\varepsilon^{\prime 2}+\varepsilon^{\prime \prime 2}}+j\frac{\varepsilon^{\prime \prime }}{\varepsilon^{\prime 2}+\varepsilon^{\prime \prime 2}}=M^{\prime }+jM^{\prime \prime }$$

where *ε′* and *Μ′*, and *ε″* and *Μ″* are the real and the imaginary part of dielectric permittivity and the electric modulus, respectively [35].

**Figure 9:**
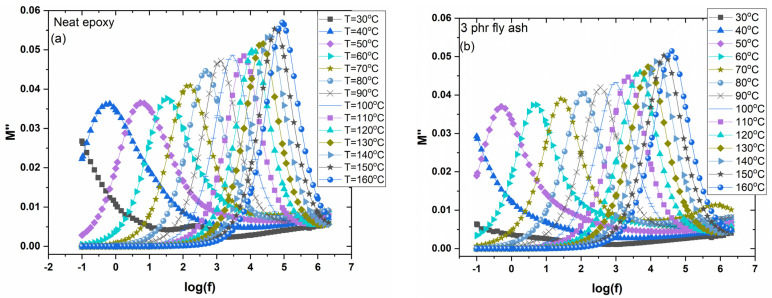
Electric modulus loss index versus frequency for (**a**) neat epoxy and (**b**) the composite with 3 phr of fly ash content, at various temperatures.

**Figure 10:**
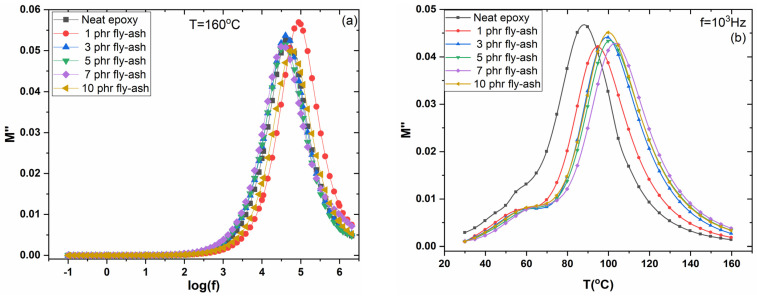
(**a**) Electric modulus loss index versus frequency, at 160 °C, and (**b**) electric modulus loss index versus temperature, at 10^3^ Hz, for all examined systems.

Figure 9a,b shows the loss modulus peaks for the unfilled epoxy matrix and the system with 3 phr of fly ash at the frequencies where transitions occur in the ε′ 3D spectra, at various temperatures. The intensive peaks are indicative of the glass-to-rubber transition. As the temperature rises, the loss peaks increase in frequency due to the frequency–temperature superposition. At temperatures between 50 and 100 °C, the rate of the main peak’s shift appears constant, while at higher temperatures, the peak shift rate decreases alongside an accompanying increase in loss modulus maxima. The shrinking of the free volume is responsible for this variation [36,37,38,39]. It is interesting to note that there is a tendency of the second peak to be formed at the high-frequency edge. This peak is attributed to β-relaxation. Figure 10a shows the frequency dependence of the imaginary part of the electric modulus (*M″*) for all studied systems at 160 °C. The formation of a loss peak indicates the presence of an α-relaxation mechanism in all samples. The peak of α-relaxation is related to the glass transition temperature (T_g_) of the amorphous polymer matrix. The thermal energy absorbed by macromolecules near T_g_ allows the cooperative segmental motion of significant portions of the polymer chains. The presence of strong or weak interactions between the polymer matrix and fillers is indicated by a shift in the loss peak to lower or higher frequencies at a constant temperature, depending on the amount and type of embedded particles [36,37,38,39,40]. Figure 10b depicts the electric modulus loss index (*M″*) as a function of temperature, at 1 kHz for all tested systems. In all spectra, the formation of two peaks is evident. The main peak is assigned to α-relaxation and its shifting toward higher temperatures, with the fly ash particles’ content, which implies an increase in the glass transition temperature, and which can be considered a result of strong adhesion/interactions between particles and macromolecules. The weaker peak recorded at low temperatures is ascribed to β-relaxation and occurs because of the rearrangement of polar side groups of the main polymer chain. The loss peak frequency’s position allows the determination of the relaxation time. Representative results are listed in Table 2. 

**Figure 11:**
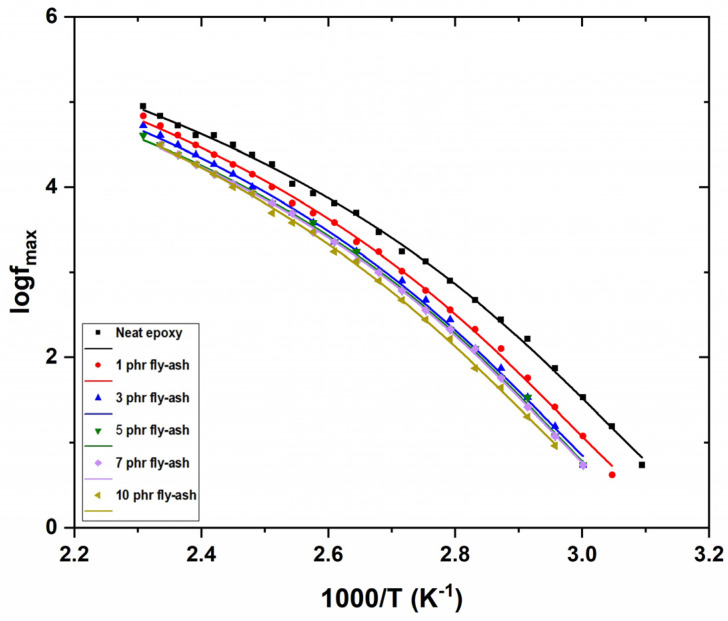
Loss peak position as a function of the inverse temperature for all examined systems, for the main loss peak.

The dynamics of α-relaxation are presented in Figure 11, where the loss peaks’ frequency, for all systems, as a function of reciprocal temperature, is depicted. As expected, the dynamics of α-relaxation deviate from Arrhenius-type behavior, because of the variation in free volume, following the Vogel–Fulcher–Tammann (VFT) relation [40]. The VFT relation is expressed by Equation (3):

(3) $$f_{max}=f_{0\cdot }exp\left(-\frac{A}{T-T_{V}}\right)$$

where *f*_0_ is a pre-exponential factor, *A* is a parameter that measures the activation energy and *T_V_* is the Vogel temperature, also referred to as the ideal glass transition temperature. Obtained dielectric data were fitted via Equation (3), resulting in the curves shown in Figure 11. Values derived from the fitting procedure, for the parameters *A* and *T_V_*, are listed in Table 2.

It is worth mentioning that both parameters increase with fly ash content indicating an increase in the glass to rubber transition temperature and in the corresponding activation energy. These findings are in accordance with the results in Figure 10 and indicative of the good adhesion/wetting of the inclusions by the matrix. 

**Figure 12:**
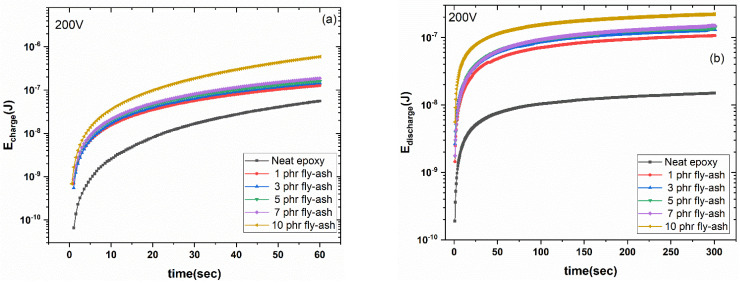

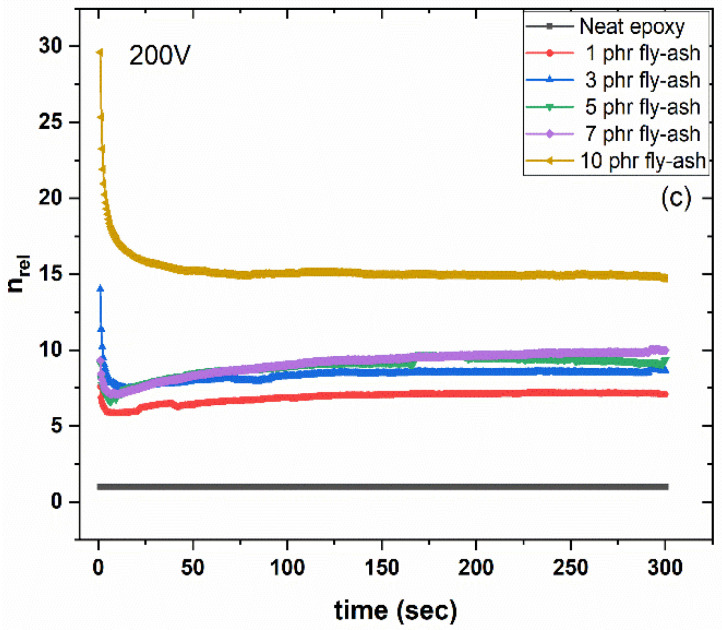
Variation in (**a**) storing energy (E_charge_), (**b**) retrieving energy (E_discharge_), and (**c**) the relative coefficient of energy efficiency (n_rel_), as a function of time, for all studied systems, at a charging voltage of 200 V.

Figure 12a,b shows the stored and retrieved energies as a function of time for all investigated systems and for a charging voltage of 200 V. Since the integrated fly ash inclusions act like a dispersed network of capacitors, both energies enhance as the filler percentage rises. This network defines a microscale active device that is capable of performing a rapid charging and discharging process for the stored/retrieved energy. Using Equation (4) and by integrating the time-dependent charge/discharge current functions, the stored and retrieved energy can be evaluated.

(4) $$E=\frac{1}{2}\frac{Q^{2}}{C}=\frac{1}{2}\frac{{\left[\int I\left(t\right)dt\right]}^{2}}{C}$$

where *E* represents the stored or retrieved energy at the composite, *Q* is the amount of stored or retrieved charge, *I*(t) is the charging or discharging current, and *C* is the capacitance of the specimen, derived from the BDS measurements at the lower frequency [24,25,41]. Due to the insulating nature of the samples, charge carriers injected by the applied voltage/electric field fail to pass through the specimens. It is possible that raising the temperature will give the carriers enough energy to overcome potential barriers, resulting in higher conductivity. At room temperature, a small fraction of charges can overcome the potential barriers, resulting in restricted charge migration and low conductivity. If a higher voltage/electric field is applied during the charge-storing procedure, potential barriers could be lowered, resulting in an increased mobility of the charges, which follow a trapping/detrapping sequence as they migrate through the extended interfacial region of the composite. Increased conductivity and leakage currents resulting from this process decrease the amount of energy recovered [24,25,41]. The introduction of the coefficient of energy efficiency (n_eff_) allows the evaluation of the storage and retrieval process. Equation (5) provides a method for determining the relative coefficient of energy efficiency:

(5) $$n_{rel}=\frac{E_{retr,\;comp}}{E_{retr,\;matrix}}$$

where *E_retr,comp_* and *E_retr,matrix_* are the retrieved energies from a composite and the matrix under the same charging voltage and at the same instance, respectively.

Figure 12c shows the time dependence of the relative coefficient of energy efficiency for the systems studied at a 200 V charging voltage. It is apparent that the ability of retrieving energy is improved with filler content and in the system with the highest fly ash content, the low-frequency edge approaches 30 times the ability of the storing/retrieving energy of the neat epoxy resin.

## 4. Conclusions

Fly ash/epoxy resin composites were prepared and studied with reinforcing phase content as a parameter. The particles were successfully integrated into the polymer matrix, as evidenced via structural and morphological characterization. Under both static and dynamic loading conditions, the thermomechanical performance of the composites was studied. The Young’s and storage moduli consistently alter as fly ash content increases. Following the change in polarization of the examined systems, the dielectric permittivity is enhanced with fly ash content and temperature, while it decreases remarkably with frequency. At low frequencies and high temperatures, IP is observed; at medium frequencies and temperatures, the glass-to-rubber transition takes place; and at high frequencies, the re-orientation of polar side groups occurs. The composite with 10 phr fly ash exhibits the optimum dielectric properties. AC conductivity values show a high scatter with both temperature and frequency. Above a critical frequency, conductivity increases exponentially with frequency, while below it, it remains roughly constant, approaching its DC value. The observed behavior is consistent with the AC universality law, which in turn suggests that hopping conduction may be involved in charge migration. Finally, the manufactured composites can be used for electrical energy storage and retrieval, with the latter ability increasing in tandem with filler content. The unique properties of these composites make them suitable engineering materials for a wide range of industrial sectors including aerospace, automotive, construction and electronics. Structural components, insulating materials, and materials for energy storing/retrieving are highlighted as potential uses for epoxy/fly ash composites. In summary, the study of epoxy/fly ash composites is a promising field for the development of sustainable and high-performance engineering materials of low cost by exploiting materials beyond their servicing life. The synergistic combination of epoxy resin and fly ash particles opens up new possibilities for engineering applications and paves the way for innovative solutions that meet performance requirements and that are environmentally sustainable.

## Figures and Tables

**Table 1:** The percentages of chemical ingredients of the employed fly ash powder.

Powder Fly Ash	%
humidity	0.00
LOI	2.50
SiO_2_	48.76
Al_2_O_3_	17.61
Fe_2_O_3_	11.12
CaO	10.86
MgO	3.09
SO_3_	2.87
K_2_O	2.08
Na_2_O	0.44
TiO_2_	0.82
Cr_2_O_3_	0.04

**Table 2:** Relaxation times for α-relaxation and values of parameters *A* and *T_V_* derived by fitting data via Equation (3).

Filler Content in Specimens (phr)	τ (s)			α-Relaxation	
	50 °C	100 °C	150 °C	*T_V_* (K)	*A* (K)
Neat epoxy	0.0294	5.424 × 10^−5^	3.026 × 10^−6^	296.79	25.35
1 phr fly ash	0.1093	9.166 × 10^−5^	3.934 × 10^−6^	302.31	26.18
3 phr fly ash	0.2401	1.549 × 10^−4^	5.114 × 10^−6^	305.58	26.46
5 phr fly ash	0.2401	1.592 × 10^4^	6.649 × 10^−6^	307.02	27.41
7 phr fly ash	0.3122	1.592 × 10^−4^	6.649 × 10^−6^	310.98	27.98
10 phr fly ash	0.5276	2.014 × 10^−4^	6.649 × 10^−6^	311.24	28.10

## Data Availability

Data are available upon request.

## Supplementary Materials

The following supporting information can be downloaded at: https://www.mdpi.com/article/10.3390/polym15163418/s1, Figure S1: Energy-dispersive X-ray spectroscopy spectrum for the composite with 1 phr fly ash. (Mapping); Figure S2: Energy-dispersive X-ray spectroscopy spectrum for the composite with 5 phr fly ash. (Mapping); Figure S3: (**a**) Energy-dispersive X-ray spectroscopy spectrum for the composite with 1 phr fly ash and (**b**) 5 phr fly ash; Figure S4: SEM image from the composite with 10 phr fly ash content, at a lower magnification; Figure S5: (**a**) Real part of dielectric permittivity, (**b**) loss tanδ, and (**c**) σ_ac_ as a function of frequency and temperature for the neat epoxy composite; Figure S6: (**a**) Real part of dielectric permittivity, (**b**) loss tanδ, and (**c**) σ_ac_ as a function of frequency and temperature for the the 5 phr fly ash/epoxy composite; Table S1: Filler content in specimens.
