# Supplementary material for: End-Of-Use Fly Ash as an Effective Reinforcing Filler in Green Polymer Composites
Source: Polymers (Basel). 2023 Aug 16;15(16):3418. doi: 10.3390/polym15163418 (PMC10459919; doi:10.3390/polym15163418)
Supplement: Supplementary file 1 [file polymers-15-03418-s001.zip › polymers-2494145-supplementary.pdf]

End of use fly ash as an effective reinforcing filler in green polymer composites

Anastasios C. Patsidis[1],* and Manolis Souliotis[2],**

[1] Department of Materials Science, University of Patras, Patras 26504, Greece,

[2] Department of Chemical Engineering, University of Western Macedonia, Kozani 50132, Greece,

* Correspondence: patsidis@upatras.gr; Tel.: +302610997835
** Correspondence: msouliotis@uowm.gr; Tel.: +302461056623

Supplementary Information

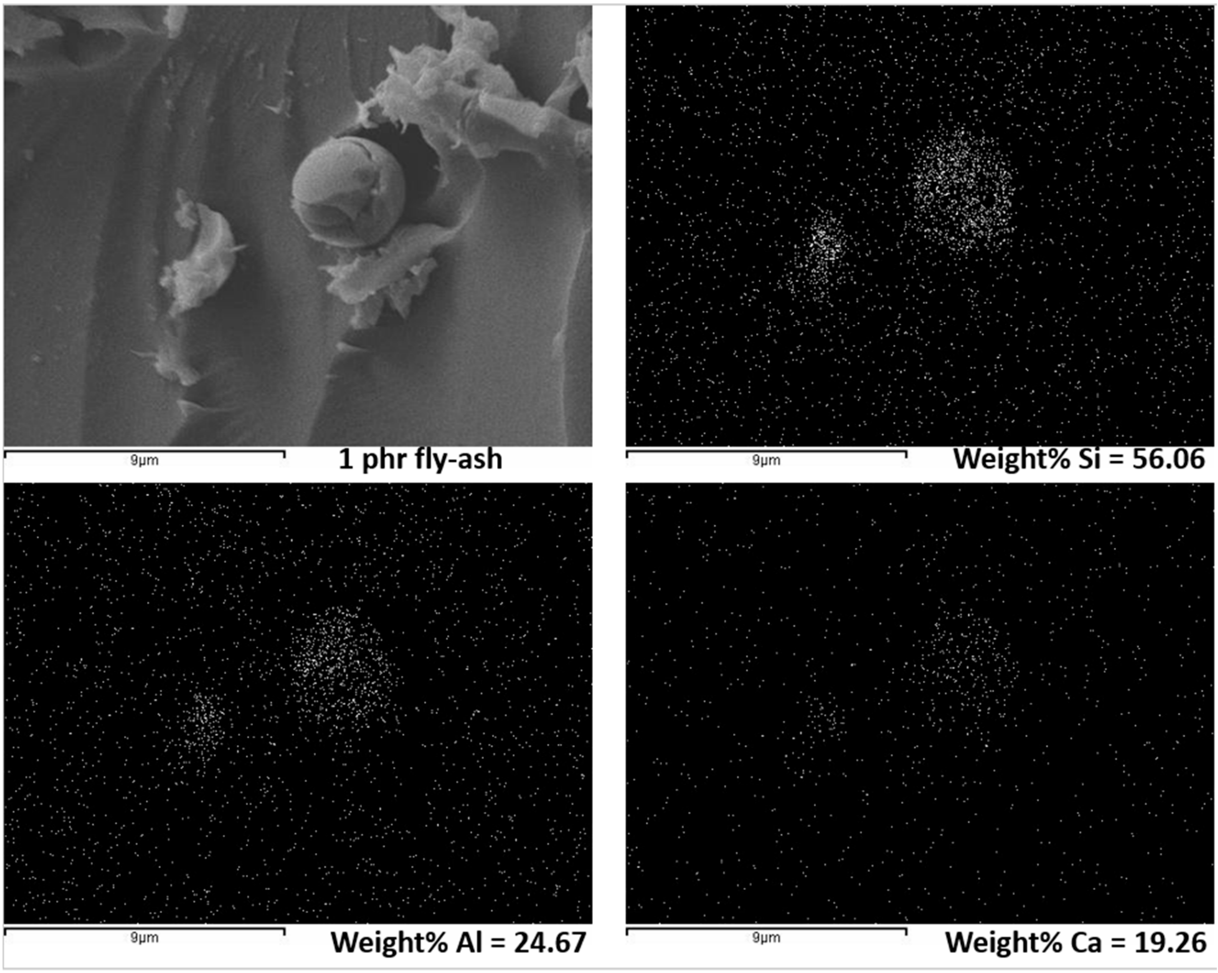

Figure S1. Energy-dispersive X-ray spectroscopy spectrum for the composite with 1 phr fly ash. (Mapping)

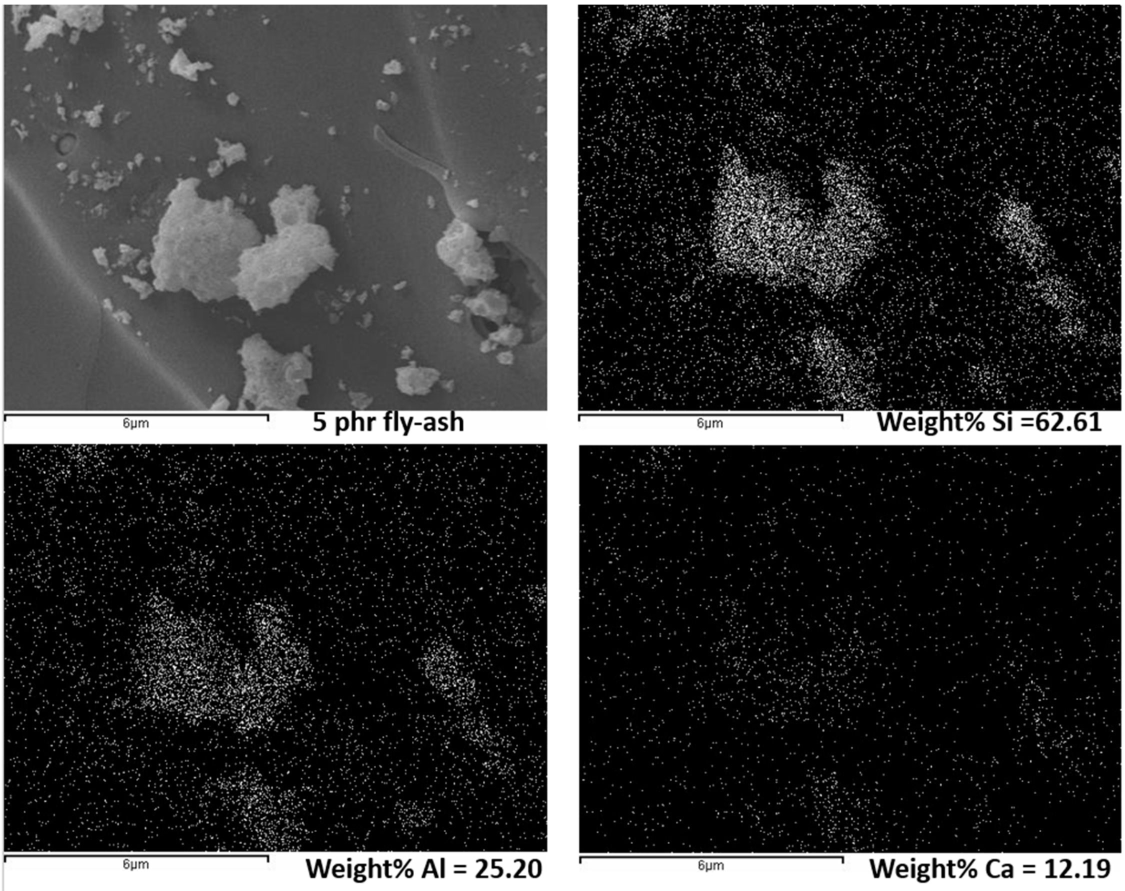

Figure S2. Energy-dispersive X-ray spectroscopy spectrum for the composite with 5 phr fly ash. (Mapping)

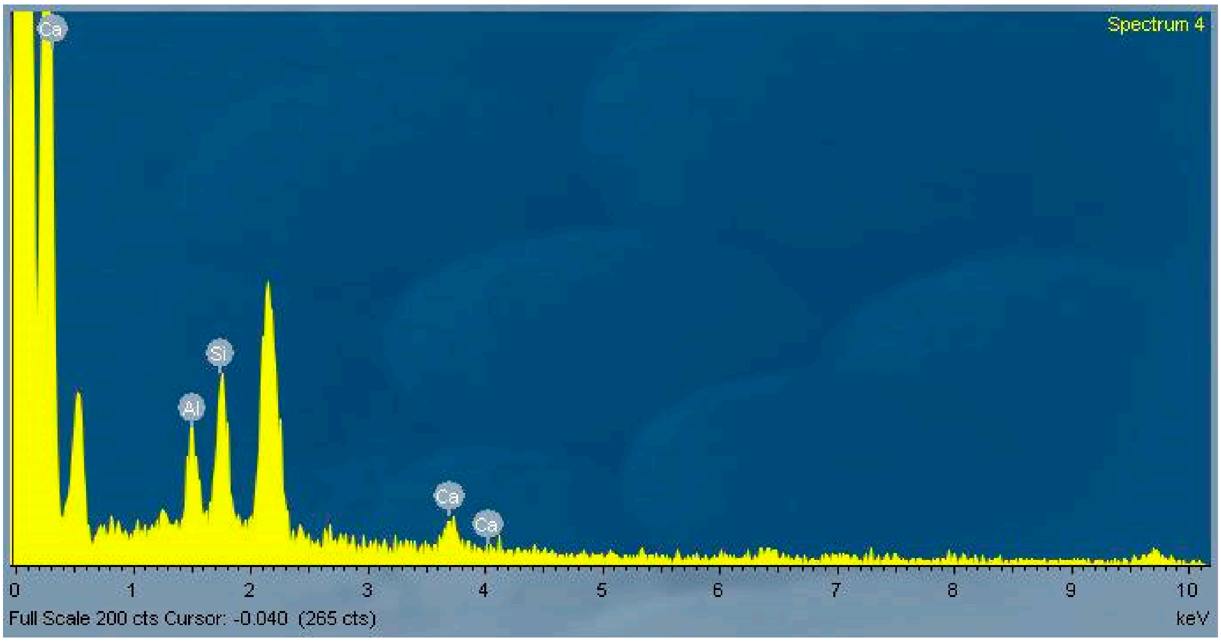

(a)

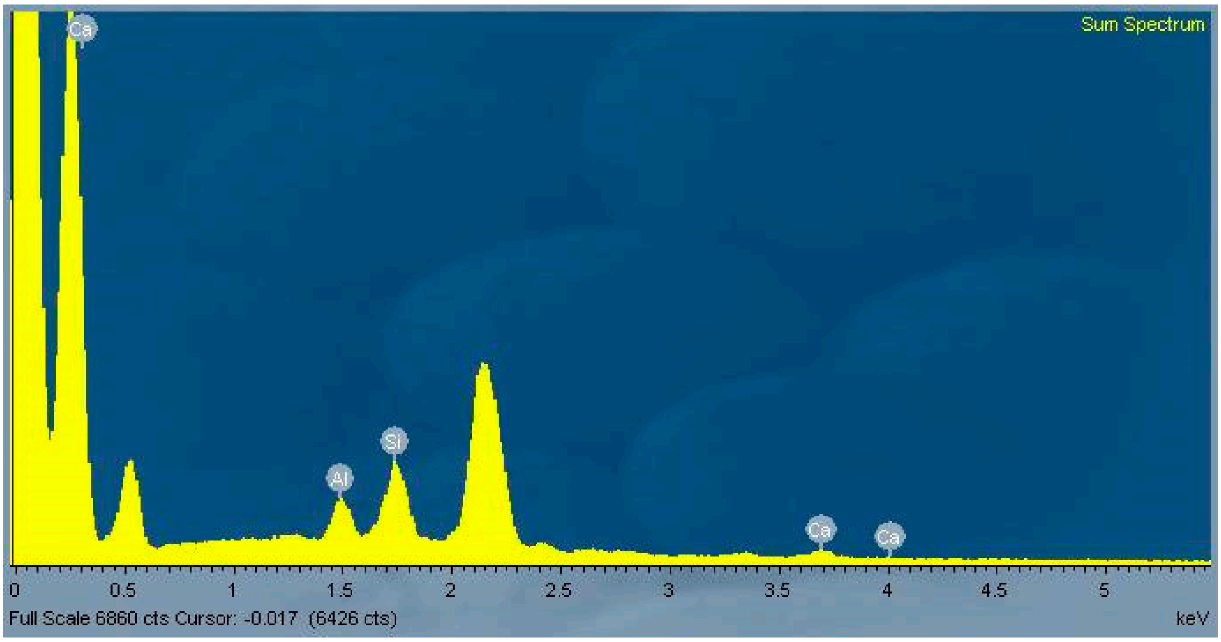

(b)

Figure S3. (a) Energy-dispersive X-ray spectroscopy spectrum for the composite with 1 phr fly ash and (b) 5 phr fly ash .

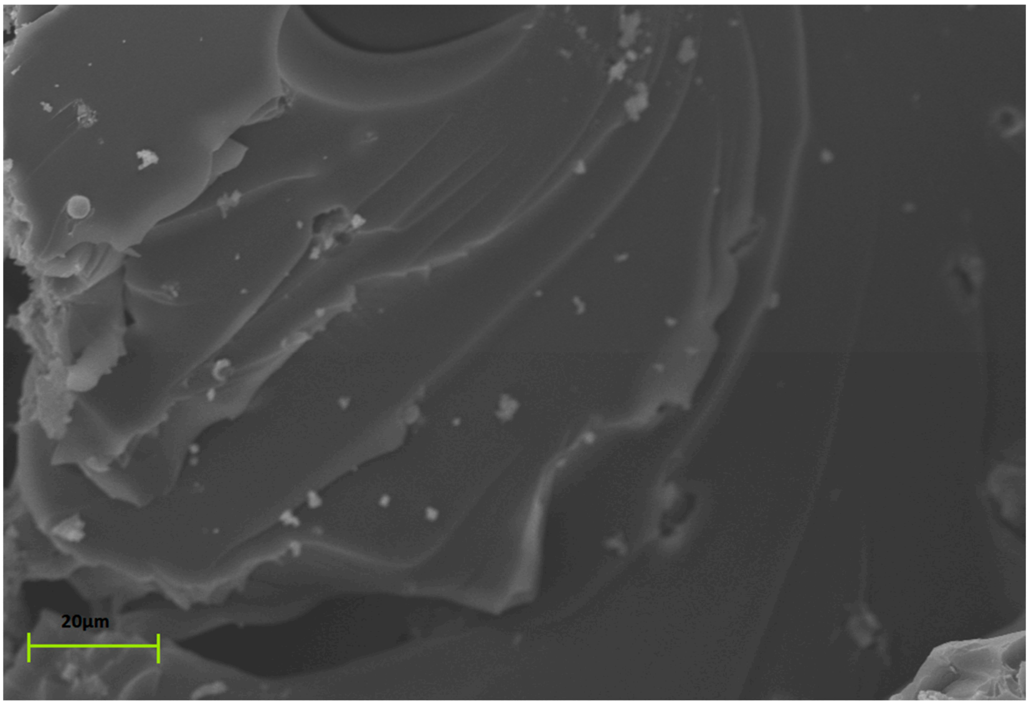

Figure S4. SEM image from the composite with 10 phr fly ash content, at a lower magnification.

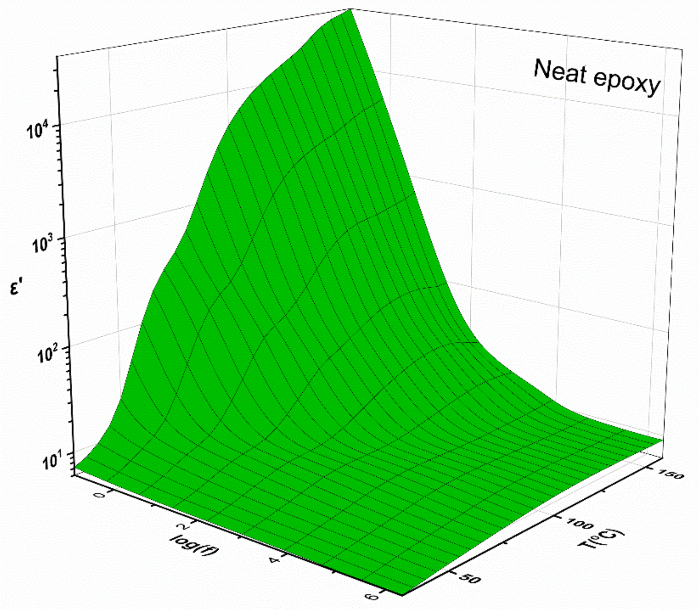

(a)

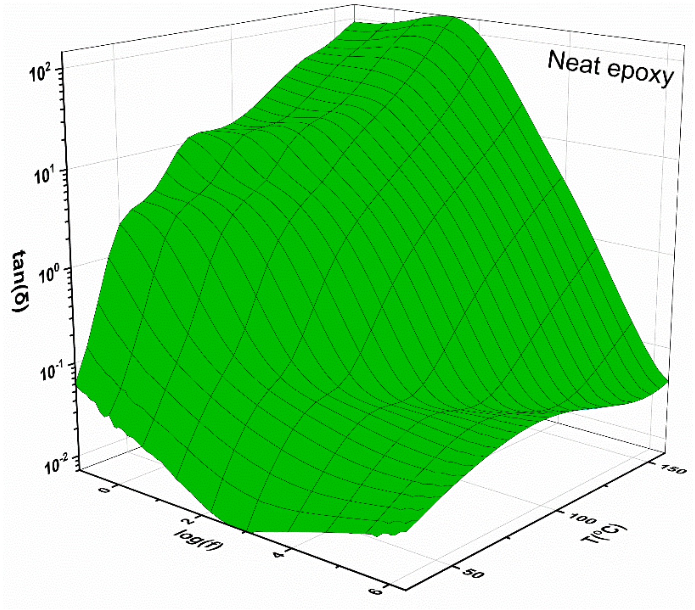

(b)

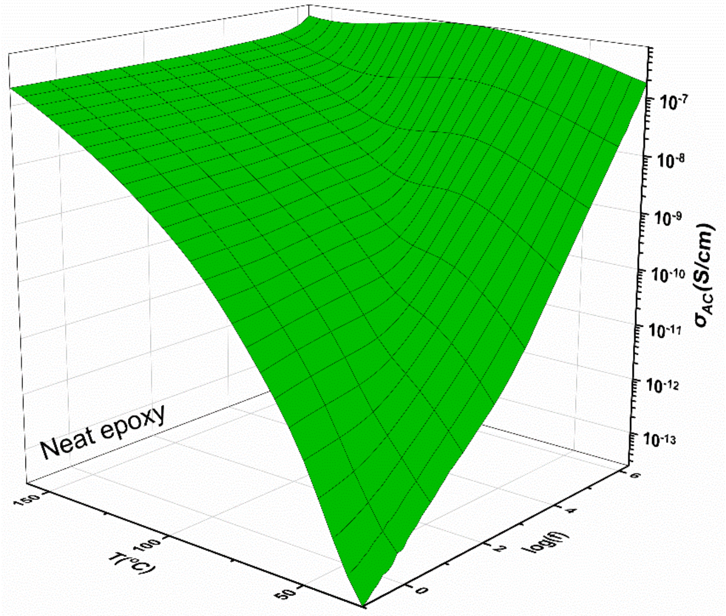

(c)

Figure S5. (a) Real part of dielectric permittivity, (b) loss tanδ, and (c) σ_{ac} as a function of frequency and temperature for the neat epoxy composite.

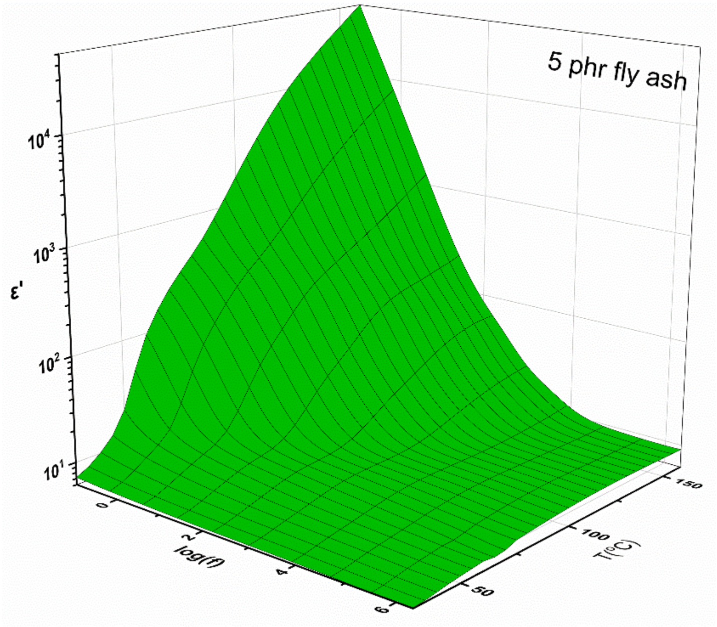
5 phr fly ash
ε'
10^4
10^3
10^2
10^1
0
2
4
6
log(f)
50
100
150
T(°C)

(a)

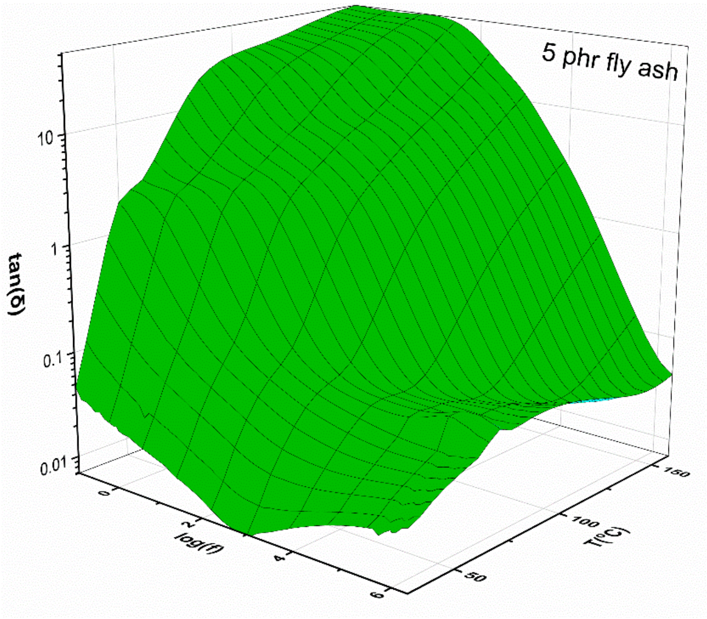
5 phr fly ash
tan(δ)
10
1
0.1
0.01
0
2
4
6
log(f)
50
100
150
T(°C)

(b)

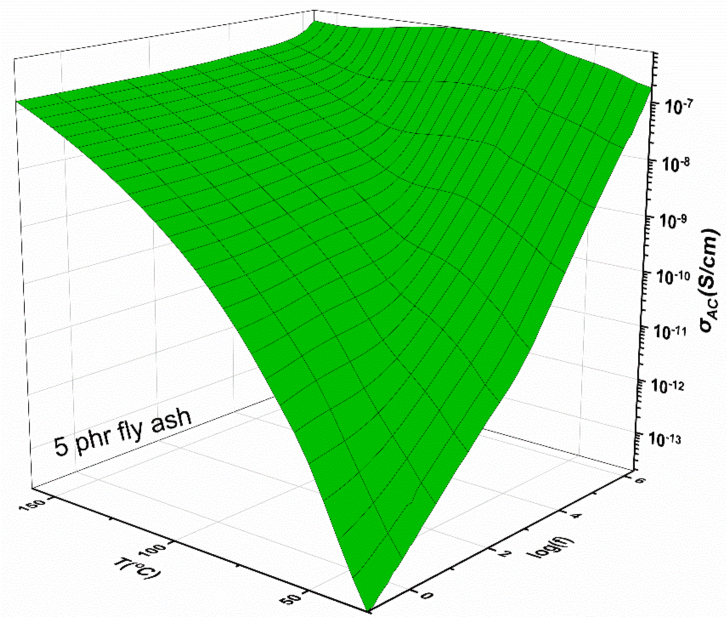

(c)

Figure S6. (a) Real part of dielectric permittivity, (b) loss tanδ, and (c) σ_{ac} as a function of frequency and temperature for the the 5 phr fly ash/epoxy composite.

Table S1. Filler content in specimens,

Filler content in specimens (phr)	Weight % Si	Al	Ca
**1 phr fly-ash**	56.06	24.67	19.26
**5 phr fly-ash**	62.61	25.20	12.19
